# Subthreshold repetitive transcranial magnetic stimulation suppresses ketamine-induced poly population spikes in rat sensorimotor cortex

**DOI:** 10.3389/fnins.2022.998704

**Published:** 2022-10-21

**Authors:** Wenxuan Jiang, Robert Isenhart, Robert Sutherland, Zhouxiao Lu, Huijing Xu, John Pace, Michael A. Bonaguidi, Darrin J. Lee, Charles Y. Liu, Dong Song

**Affiliations:** ^1^Department of Biomedical Engineering, University of Southern California, Los Angeles, CA, United States; ^2^Rancho Los Amigos National Rehabilitation Center, Downey, CA, United States; ^3^Neurorestoration Center, University of Southern California, Los Angeles, CA, United States; ^4^Department of Neurological Surgery, University of Southern California, Los Angeles, CA, United States

**Keywords:** rTMS, ketamine, local field potentials, population spikes, spikes, neuromodulation

## Abstract

Cortical oscillations within or across brain regions play fundamental roles in sensory, motor, and memory functions. It can be altered by neuromodulations such as repetitive transcranial magnetic stimulation (rTMS) and pharmacological manipulations such as ketamine. However, the neurobiological basis of the effects of rTMS and ketamine, as well as their interactions, on cortical oscillations is not understood. In this study, we developed and applied a rodent model that enabled simultaneous rTMS treatment, pharmacological manipulations, and invasive electrophysiological recordings, which is difficult in humans. Specifically, a miniaturized C-shaped coil was designed and fabricated to deliver focal subthreshold rTMS above the primary somatosensory (S1) and motor (M1) cortex in rats. Multi-electrode arrays (MEA) were implanted to record local field potentials (LFPs) and single unit activities. A novel form of synchronized activities, poly population spikes (PPS), was discovered as the biomarker of ketamine in LFPs. Brief subthreshold rTMS effectively and reversibly suppressed PPS while increasing the firing rates of single unit activities. These results suggest that ketamine and rTMS have convergent but opposing effects on cortical oscillations and circuits. This highly robust phenomenon has important implications to understanding the neurobiological mechanisms of rTMS and ketamine as well as developing new therapeutic strategies involving both neuromodulation and pharmacological agents.

## Introduction

Cortical oscillations within or across multiple brain regions play fundamental roles in sensory (Engel and Singer, 2001; Fries et al., 2001; Buzsaki and Draguhn, 2004), motor (Farmer, 1998; Davis et al., 2012; van Wijk et al., 2012), and memory functions (Lisman, 2010; Nyhus and Curran, 2010). Prominent rhythmic oscillations have been classically divided into delta (1–4 Hz), theta (4–8 HZ), alpha (8–12 Hz), beta (12–35 Hz), and gamma (35–100 Hz) bands. Since endogenous cortical oscillations are often disrupted in neurological and neuropsychiatric conditions, they are often used as biomarkers in diagnosis and treatment of these diseases (Popovych and Tass, 2014; Rabiller et al., 2015).

Cortical oscillations can be altered by neuromodulation and pharmacological manipulations. For example, in repetitive transcranial magnetic stimulation (rTMS), a non-invasive neuromodulation procedure, an external TMS coil is used to induce a changing electromagnetic field to activate or inhibit the nervous system (Barker, 1991; Hallett, 2007). Although the underlying mechanisms remains largely unknown, it has been reported that rTMS can effectively modulate cortical oscillations in different frequency bands with different stimulation parameters (Paus et al., 2001; Ding et al., 2014; Zmeykina et al., 2020; Yang et al., 2021). Ketamine, a medication originally used for anesthesia and analgesia (McCarthy et al., 1965; Meyer and Fish, 2008; Mazzeffi et al., 2015; Vadivelu et al., 2016), has drawn much attention recently for its use as an antidepressant and a psychedelic agent (Krystal et al., 1994, 2019; Steeds et al., 2015; Andrade, 2017; Ballard and Zarate, 2020). It has been shown that ketamine decreases powers of neural oscillations in low frequency (delta, theta, and alpha) bands while increasing power in high frequency (gamma) band at both anesthetic and subanesthetic doses (Hong et al., 2010; Chauvette et al., 2011).

Due to their overlapping effects, rTMS and subanesthetic ketamine have been combined to treat neuropsychiatric disorders in humans and shown synergetic effects (Best and Griffin, 2015; Pradhan and Rossi, 2020; Davila et al., 2021). However, the interaction of rTMS and anesthetic ketamine on cortical activities including cortical oscillations and neuronal spike firing has not been explored yet. To understand the neurobiological basis of the effects of rTMS and ketamine as well as their interactions on cortical oscillations, in this study, we developed and applied a rodent model that enabled simultaneous rTMS treatment, pharmacological manipulations, and invasive electrophysiological recordings, which is difficult or impossible in human studies.

Specifically, a miniaturized TMS coil was designed, fabricated, and characterized for rodent brain stimulation. This coil can deliver focal subthreshold TMS to the primary somatosensory (S1) and motor (M1) cortex in rats. Multi-electrode arrays (MEA) were also implanted in the S1 and M1 to record both local field potentials (LFPs) and single unit activities. Using this rodent model, we discovered a novel form of synchronized activities, i.e., poly population spikes (PPS), as the biomarker of ketamine in LFPs. Such activities could be highly reliably induced by ketamine. More intriguingly, we found that brief (3-min duration) subthreshold rTMS can effectively and reversibly suppress PPS while increasing the firing rates of spontaneous single unit activities. These results demonstrated that ketamine and rTMS have convergent but opposing effects on cortical oscillations and circuits. This highly robust phenomenon may have important implications to understanding the neurobiological mechanisms of rTMS and ketamine as well as developing new therapeutic strategies involving both neuromodulation and pharmacological agents.

## Materials and methods

### Miniaturized TMS coil

**FIGURE 1:**
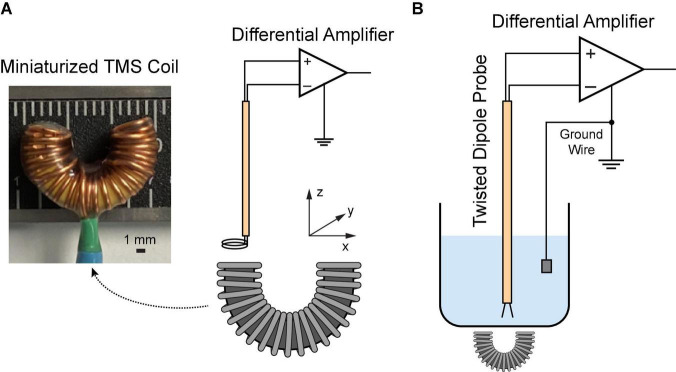
Characterization of the miniaturized C-shaped coil. **(A)** Measure magnetic field *via* a two-loop search coil. **(B)** Measure induced electric field *via* a dipole probe in saline.

As previously described (Jiang et al., 2021), a miniaturized TMS coil (5 mm outer diameter) was built with a C-shaped iron powder core (Figure 1). Insulated copper wires were wound (30 turns) around the core and evenly distributed over its circumference. The coil was coated with a layer of silicone (422C, MG Chemicals, Canada) for extra insulation. Like a conventional figure-eight coil, the C-shaped coil generates a strong, focal electric field in the middle that is induced by opposing electrical currents from both ends of the coil. The core material (Micrometals, Anaheim CA, USA) composed of insulated iron powder provides high permeability and high saturation flux density, inducing a stronger electric field.

A customized MATLAB script was utilized to create the stimulation pattern, which was input into an Arbitrary Waveform Generator (Agilent 33500B Series, Santa Rosa CA, USA) to control the stimulation parameters. The custom waveform is a Gaussian pulse with a peak amplitude at 7.5 V and standard deviation at 30 μs. A capacitor array was used to generate the current pulses in the coil.

### Magnetic field measurement

A two-loop search coil with a diameter of 2.3 mm was used to measure the varying magnetic flux (Meng et al., 2018). The search coil was positioned at different coordinates above the TMS coil (Figure 1A). The signal was amplified *via* a Model 1,700 differential AC amplifier (A-M systems, Sequim WA, USA) with a gain of 100. The induced voltages were measured with a Digidata 1322A recording equipment (Molecular Devices, Sunnyvale CA, USA). Based on the Faraday’s law of induction, the induced voltage *V* is given by


$$V=-N\,\frac{d\,\phi_{B}}{d\,t}$$

Where *N* is the number of turns and *ϕ*_*B*_ is the magnetic flux through the search coil. Assuming that the induced magnetic field remains homogenous over the loop, the magnetic field *B* in different directions can be estimated as


$$B_{x}=-\frac{1}{N\,r^{2}\,\pi }\,\int V_{x}\cdot dt,B_{y}=-\frac{1}{N\,r^{2}\,\pi }\,\int V_{y}\cdot dt,B_{z}\,=-\frac{1}{N\,r^{2}\,\pi }\,\int V_{z}\cdot dt$$

where *V_x_*, *V*_*y*_, and *V_z_* represent the induced voltages measured along *x*, *y*, and *z* directions, respectively; *r* is the radius of the search coil.

### Electric field measurement

In order to reduce the electromagnetic interference during the electric field measurement, a dipole probe was fabricated by twisting two insulated copper wires (Tofts and Branston, 1991; Mueller et al., 2014). It has a 3-mm separation at the exposed ends. Saline solution (0.9% NaCl) was filled in a 1,000 mL glass container. The TMS coil was put underneath the container. During stimulation, the dipole probe was positioned at different coordinates in saline (Figure 1B). The signal was amplified 1,000 times by a DAM50 differential amplifier (World Precision instruments, Sarasota FL, USA); and it was measured with the Digidata equipment mentioned as above. Considering the linearity of the electric field between two closely spaced points (Tofts and Branston, 1991), the electric field in each direction can be approximated as


$$E_{x}=-\frac{△\,V}{△\,x},E_{y}=-\frac{△\,V}{△\,y},E_{z}=-\frac{△\,V}{△\,z}$$

where *E_x_*, *E*_*y*_, and *E_z_* are induced electric fields in each direction; △*V* is the amplitude of the first peak of recorded waveforms; △*x*, △*y*, and △*z* are the known distances (3 mm).

### Pharmacological manipulations and electrophysiological recording

**FIGURE 2:**
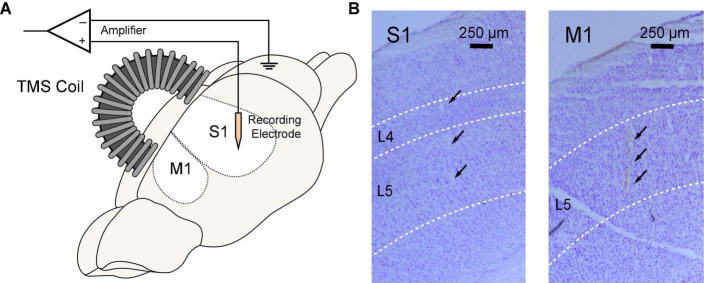
Simultaneous rTMS and intracranial electrophysiological recording in rat sensorimotor cortex. **(A)** TMS coil was positioned above the skull and parallel to the midline with an incidence angle of 15^°^. A 64-channel silicon probe was implanted to the primary somatosensory (S1) or motor cortex (M1). **(B)** Locations of silicon probes were verified in brain slices with cresyl violet (Nissl) staining. Probe tracks were identified at layer 4–5 of the S1 (left) or layer 5 of the M1 (right).

All animal experiments were conducted following protocols approved by the Institutional Animal Care and Use Committee of the University of Southern California. Sprague-Dawley rats (*n* = 11, female, 220–250 g, 11–12 weeks) were used in this study. Animals underwent a rapid inhaled induction with 4% isoflurane, followed by an intraperitoneal injection of ketamine (75 mg/kg) and xylazine (10 mg/kg) mixture. Additional doses of ketamine (36 mg/kg) were injected intraperitoneally to maintain a constant anesthesia level, which was evaluated *via* the hindlimb pedal withdrawal reflex and breathing rate. Body temperature was maintained with a feedback-controlled heating pad. Rats were mounted on a stereotaxic frame *via* ear bars and a nose cone in a Faraday cage. A right-sided craniotomy was conducted to expose the S1 and M1. Dura mater was removed. A 64-channal silicone probe (Neuronexus A8 × 8-Edge-5 mm-50-150-177, Ann Arbor MI, USA) was implanted in layer 5 of the M1 or layer 4–5 of the S1 using a micromanipulator (Figure 2). The probe was parallel to the sagittal suture (midline) of the skull. The stereotaxic coordinates of the implantation sites relative to the bregma are shown in Table 1. Two small holes were drilled on the occipital bone to place the ground wire and the reference wire.

**TABLE 1:** Targeted cortex, corresponding stereotaxic coordinates of the first shank, and unit yields of animal used in this study.

Animal ID	Cortex	Stereotaxic coordinates			Unit yield
		AP	ML	Depth	
Rat01	S1	0.00	3.80	–2.00	46
Rat02	S1	0.00	3.80	–2.59	29
Rat03	S1	0.00	3.80	–2.12	21
Rat04	S1	0.00	3.80	–2.05	34
Rat05	S1	0.00	3.80	–2.30	33
Rat06	M1	2.40	3.00	–2.00	28
Rat07	M1	2.40	3.00	–2.10	19
Rat08	M1	2.40	3.00	–2.14	44
Rat09	M1	2.40	3.00	–2.49	31
Rat10	M1	2.40	3.00	–2.11	27
Rat11	S1	0.00	3.80	–2.10	–

S1, primary somatosensory cortex; M1, primary motor cortex; AP, anterior-posterior; ML, medial-lateral. Unitary activity was not recorded in Rat11.

The TMS coil was parallel to the midline and tilted at 15^°^ to ensure that its center point was right above the electrode position (Figure 2). The coil was placed above the skull with a distance of ∼1 mm. Two courses of 3-min, 10 Hz rTMS (∼1,800 pulses) were delivered to the S1 or M1 with a 15-min interval. LFPs and spontaneous unitary (spiking) activities were monitored before and after each course of rTMS in all animals. Five minutes of recordings prior to each stimulation was used as baseline. To keep the consistency of electrophysiological recordings, the experiment was restricted to a 45-min window when the animal was under a stable anesthesia depth. Wideband signals were acquired with an OmniPlex Neural Recording Data Acquisition System (Plexon, Dallas TX, USA) at a 40 kHz sampling rate.

Spike sorting was performed with a Plexon offline sorter. Only units with a clear refractory period, an above 50 μV peak-to-peak amplitude, and a consistent waveform shape were included in the analysis. Frequency and time-frequency analysis was performed with a custom MATLAB script. The data was first lowpass filtered and downsampled to 1,000 Hz. To visualize the frequency characteristics across the continuous recording, the Welch’s method was used to compute the power spectral density (power spectrum). Time-frequency data was visualized *via* the short-time fast Fourier transform (FFT) using a Hann window of 1 s with 75% overlap. The resulting output was a spectrogram with a temporal resolution of 0.25 s and a frequency resolution of 1 Hz.

A single silicon probe was utilized throughout the study. The electrode impedance was re-measured *in vitro via* a NanoZ impedance tester (White Matter, Seattle WA, USA) after the experiment. All functional electrodes remained at an impedance of about 0.5 MΩ at 1 kHz. The seventh shank (Site49-57) of the probe was broken in the recordings of Rat04 to Rat11. Signals recorded from those unfunctional channels were not included in analysis. Recordings in Rat03 were 5-min shorter than recordings in other animals.

After the experiment, animals were perfused transcardially with 10% formalin. The rat brain was extracted from the skull, dehydrated in 18% sucrose, stored at 4^°^C, and then sliced into 50-μm-thick coronal brain slices with a Cryostat (Leica, Buffalo Grove IL, USA). To verify recording locations, brain slices were stained with cresyl violet and photographed under a microscope (Leica, Buffalo Grove IL, USA).

### Statistical analysis

All data are presented as the mean ± standard error of the mean (SEM). A two-tailed *t*-test was performed to compare the normalized power of LFP bands for each minute after rTMS to the 5-min pre-rTMS baseline (significance level: α = 0.05). A two-tailed *t*-test was performed to compare the normalized PPS features for each minute after rTMS to the 5-min pre-rTMS baseline (significance level: α = 0.05). A paired *t*-test was conducted to compare the pre-rTMS and post-rTMS neuronal firing rates (significance level: α = 0.01).

## Results

### TMS coil characterization

**FIGURE 3:**
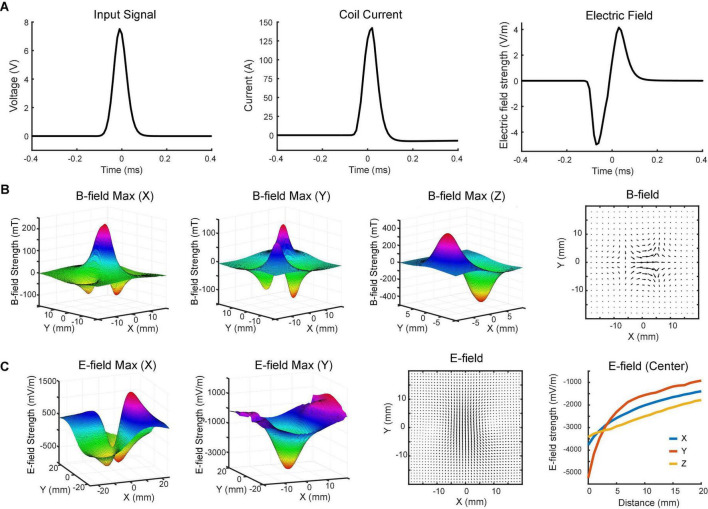
Magnetoelectric fields induced by the TMS coil. **(A)** Input signal to the coil (left), coil current (middle), and induced biphasic voltage waveform (right) measured on benchtop and in saline. **(B)** x-, y-, and z-components of the magnetic field measured with the search coil at maximum intensity (z = 0). The total magnetic field in the horizontal (x-y) plane is depicted in the vector plot. **(C)** x- and y-components of the electric field measured in saline with the dipole probe positioned 4 mm above the coil at maximum intensity. The total electric field in the horizontal plane is depicted in the vector plot. x-, y-, and z-components of electric field at different distances (0–20 mm) were measured at the center of the coil.

Before animal experiments, we first characterized the TMS coil on the benchtop and in saline. Input pulse to the coil had a Gaussian waveform that was generated with an Arbitrary Waveform Generator (Agilent 33500B Series, Santa Rosa CA, USA). It had a maximum strength at 7.5 V and standard deviation at 30 μs (Figure 3A). At 100% stimulator output, the coil current showed a peak amplitude at 142 A measured across a 0.05 Ω resistor (Figure 3A). The transient current induced a biphasic voltage waveform in saline (Figure 3A).

The magnetic field was measured in a 3D space above the coil surface in the air *via* a search coil (Figure 1A). It had a resolution of 2 mm along each direction. Figure 3B shows the measured strength of the x-, y-, and z-components of magnetic field, respectively. The maximum strength of x-component was measured at the center of the coil. Another two peaks were detected on the two sides of the coil with an opposite polarity. The minimum strength of y-component was detected near the center point, while maximum strengths were measured in the four quadrants. These peaks showed opposite polarity at the adjacent quadrants but same polarity at the diagonal quadrants. The results indicated that z-direction was the dominant component of the magnetic field near the coil surface. At z = 0 mm, the strongest strengths were detected at two ends of the coil. The north pole had a peak amplitude at 406 mT, while the south pole had a peak amplitude at –473 mT. The slight difference between two poles was caused by the uneven surface of the hand-made magnetic core.

To further characterize the coil, we measured the electric field induced in the horizontal (x-y) plane *via* a dipole probe (Figure 1B). Since the thickness of the glass is ∼3.5 mm, the dipole probe was positioned 4 mm away from the coil surface. The electric field measurements had a resolution of 1 mm along each direction. Figure 3C illustrates the x- and y-components of electric field induced in the horizontal plane. The minimum strength of x-component was detected near the center point, while maximum strengths were captured in the four quadrants. Similar to the y-component of magnetic field, those peaks showed opposite polarity at the adjacent quadrants but same polarity at the diagonal quadrants. The maximum strength of y-component was about 5.2 V/m in the middle, where the x-component was almost zero. It was due to the opposite currents at both ends of the coil reinforcing the y-component and counterbalancing the x-component of electric field at the center.

The overall magnetic and electric fields in the horizontal plane were depicted in vector plots (Figures 3B,C). The length of the arrow denotes the field strength, while the direction of the arrow indicates the direction of the field. An electric field with ring shapes was induced in the horizontal plane (Figure 3C). Along the z-direction, the induced electric field was measured at various depths. Results showed that the field strength rapidly decreased within 10 mm (Figure 3C).

### Ketamine induces poly population spikes in addition to slow-wave activities

**FIGURE 4:**
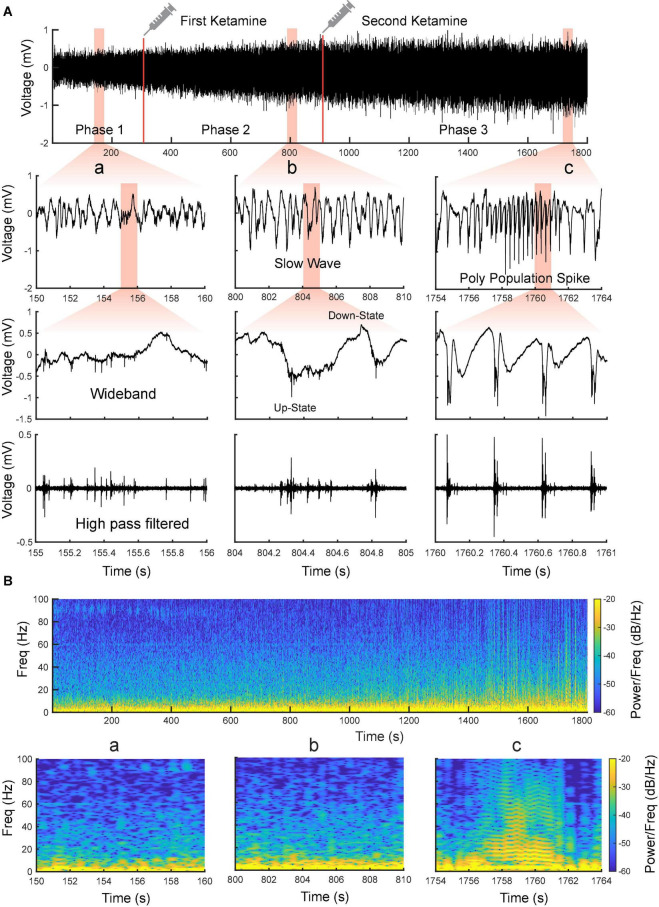
Evolvement of sensorimotor cortical signals with ketamine injections. **(A)** Intracranial recording (S1, Rat11) are divided into three phases by two doses of ketamine. Representative data segments from the three phases are expanded to different timescales **(a–c)**. a Low-amplitude activity before the first additional dose of ketamine. b Slow-wave activity after the first additional dose of ketamine. c Poly population spikes (PPS) after the second additional dose of ketamine. These segments are further expanded and high pass filtered (250 Hz) to show the correlation between different states of LFP and neuronal spike firing (fourth row). **(B)** Spectrograms of the signals shown in **(A)**.

**FIGURE 5:**
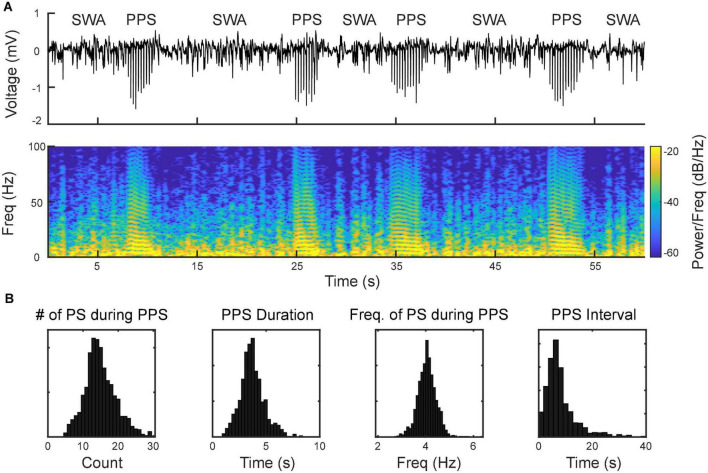
Alternation between slow-wave activities (SWA) and PPS as the signature pattern of ketamine. **(A, top)** Wideband signal showing SWA and PPS; **(A, bottom)** spectrogram of the signal. The horizontal strips during PPS reflect the harmonics of the fundamental frequency of population spikes within PPS. **(B)** Distributions of number of population spikes (PS) during PPS, PPS duration, frequency of PS during PPS, and PPS-PPS intervals.

Using the MEA, we monitored electrophysiological activities from the S1 and M1 *in vivo* in rats (Figure 2). Neural activities recorded after anesthetic dosing of ketamine are divided into three phases (Figure 4). The first phase started right after MEA implantation, which happened approximately 40 min after the first ketamine/xylazine administration. This phase was close to the recovery from anesthesia. It showed low-amplitude activities in the LFP and continuous neuronal firing (Figures 4Aa,Ba). When the animal had a positive pedal withdrawal reflex, the first additional dose of ketamine was administered, which initiated the second phase. Slow-wave activities (SWA) were rapidly induced by ketamine, which was evident by the increasing amplitude of LFP and the periodic patterns of spiking activity (Figures 4Ab,Bb). This phenomenon was reported in several previous studies (Chauvette et al., 2011; Fiáth et al., 2016; Neske, 2016; Horváth et al., 2021). Two states alternated in SWA: up-states with intensive neuronal spike firing and down-states with cessation of neuronal spike firing. As the animal was still at a light plane of anesthesia, the second additional dose of ketamine was administered 10 min after the first additional dose to start the third phase. After the second additional dose, the amplitude of LFP further increased, and the neuronal spike firing became highly rhythmic. Most interestingly, a novel LFP pattern was observed: within a few minutes of the second additional dose, a train of high-voltage (> 1 mV) population spikes, i.e., PPS started to appear. The PPS pattern was irregular at first. Ten minutes after the second additional dose, while the animal was under deep anesthesia without any reflexes, the PPS pattern gradually became more regular until it reached a stable state, characterized as a train of 10–20 population spikes with a 50–100 ms duration and a 3–5 Hz frequency lasting for 3–5 s (Figures 4Ac,Bc, 5). The PPS then alternated with the SWA (Figure 5A). Like neuronal spike firings during SWA (up-states), the neuronal spike firing occurred during depth-negative phases of PPS. This ketamine-induced PPS persisted for > 45 min across the cortex, and it was synchronous across all recording channels.

### Repetitive transcranial magnetic stimulation suppresses ketamine-induced poly population spikes and changes the power of local field potential bands

**FIGURE 6:**
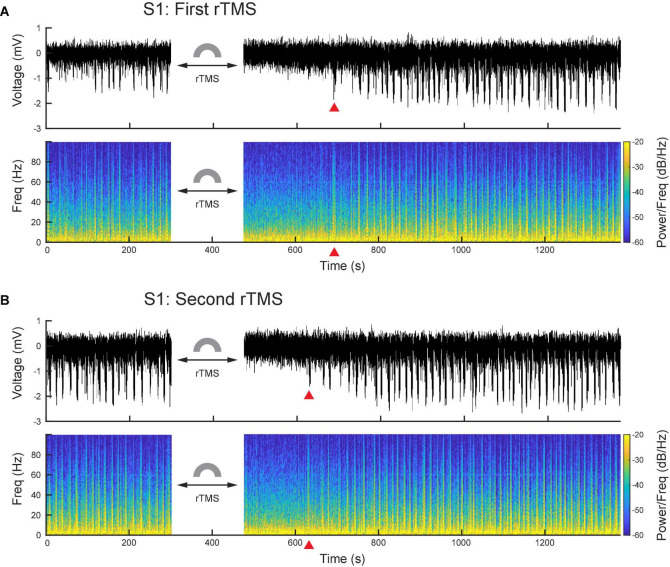
PPS were effectively and reversibly suppressed by rTMS. **(A)** Wideband signal and its spectrogram before and after the first course of rTMS. **(B)** Wideband signal and its spectrogram before and after the second course of rTMS. Red triangles denote the first PPS occurred after rTMS.

After PPS were induced, we further applied two courses of rTMS (10 Hz, 3 min) to the cortices and monitored electrophysiological activities continuously. Compared with the baseline recording, rTMS effectively and reversibly suppressed the ketamine-induced PPS after both courses of stimulation (Figures 6A,B). In the first course of rTMS, no PPS were observed after the end of rTMS until 219 ± 16 s later in the S1 (*n* = 5) and 181 ± 14 s later in the M1 (*n* = 5). In the second course of rTMS, the PPS reappeared 153 ± 10 s in the S1 (*n* = 5) and 85+13 s in the M1 (*n* = 5) after the end of rTMS.

**FIGURE 7:**
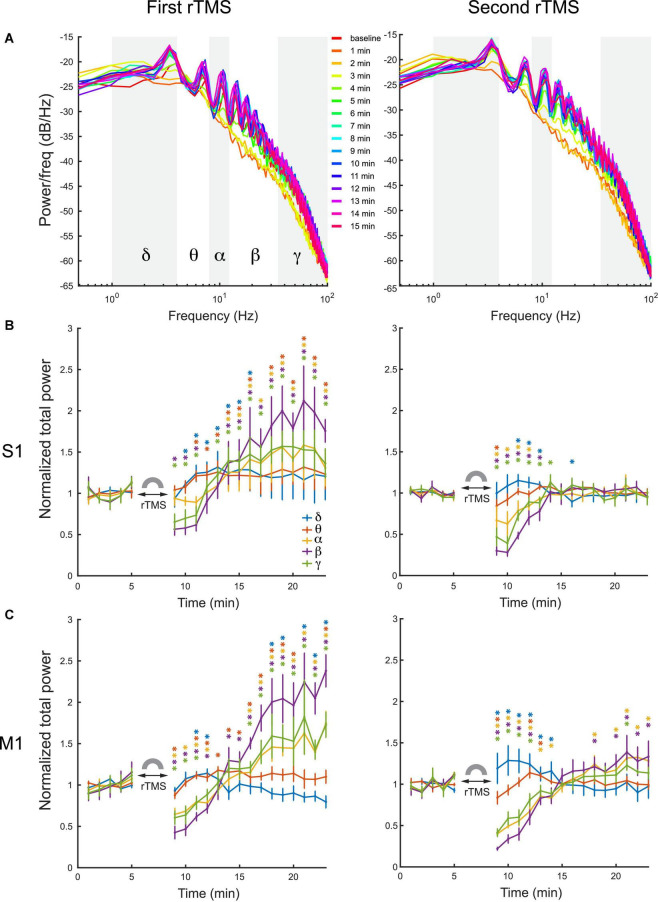
Changes of LFP power distribution in different frequency bands in the S1 and M1 after each course of rTMS. **(A)** Power spectra at every minute before and after two courses of rTMS (Rat01). The mean power of delta (1–4 Hz), theta (4–8 HZ), alpha (8–12 Hz), beta (12–35 Hz), and gamma (35–100 Hz) bands are calculated and compared with the baseline level (*p < 0.05, two-tailed *t*-test). **(B)** The mean power of each frequency band in the S1 before and after each course of stimulation (*n* = 5). **(C)** The mean power of each frequency band in the M1 before and after each course of stimulation (*n* = 5). Error bars: SEM.

Quantitative analysis was conducted on power spectra of LFPs with 1-min resolution (Figure 7A). LFP frequency was classified into delta (1–4 Hz), theta (4–8 HZ), alpha (8–12 Hz), beta (12–35 Hz), and gamma (35–100 Hz) bands in this analysis. Mean power of each individual frequency band was computed for each course of rTMS. Results showed that the first course of rTMS significantly decreased the beta and gamma power of the LFP in the S1, while there was an increase in all power bands after ∼5 min, compared to the baseline (Figure 7B). In the second course of rTMS in the S1, there was a significant suppression in theta, alpha, beta, and gamma band powers, while there was an enhancement in the delta band power (Figure 7B). Different from the first course of rTMS, the mean power of each frequency band returned to the baseline level ∼5 min after the second course of rTMS. In the M1, the mean power of LFP had similar results to those observed in the S1. rTMS significantly decreased the theta, alpha, beta, and gamma band powers in both courses of stimulation, while the delta band power increased in the second course of rTMS (Figure 7C). The effect of rTMS persisted for ∼5 min in both courses. After that, there was an increase in alpha, beta, and gamma band powers after the first course of stimulation. However, the mean power of each band returned to the baseline level after the second course of stimulation. Those results indicated that rTMS modulated the neural oscillations in different frequencies in both S1 and M1.

**FIGURE 8:**
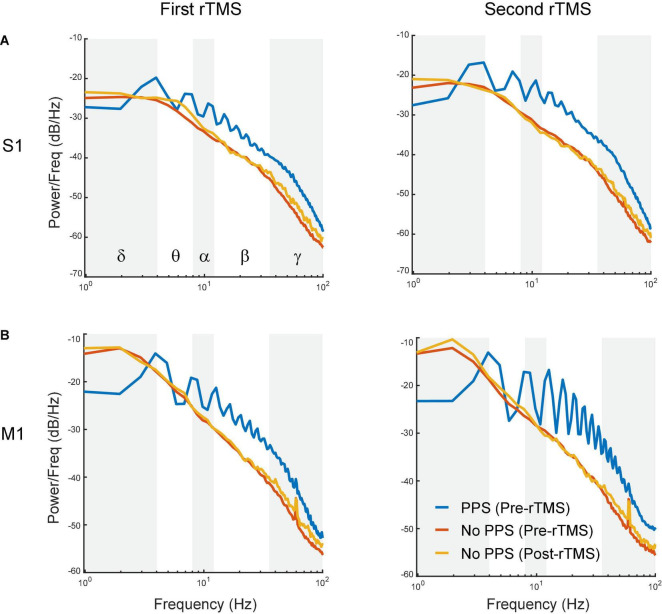
Comparison of power spectra between pre-rTMS LFP during PPS (blue), pre-rTMS LFP without PPS (red), and post-rTMS LFP without PPS (yellow). **(A)** The first (left) and second (right) course of rTMS in the S1. **(B)** The first (left) and second (right) course of rTMS in the M1. Note the signature “sawtooth” pattern in PPS.

To further assess whether the suppression of PPS by rTMS alone could explain the changes in powers of frequency bands caused by rTMS, three types of signals, i.e., pre-rTMS LFP during PPS, pre-rTMS LFP without PPS, and post-rTMS LFP without PPS, were separated from the data, and their power spectra were compared (Figure 8). Pre-rTMS LFP during PPS and pre-rTMS LFP without PPS signals were obtained from 5 min before rTMS. Post-rTMS LFP without PPS signals were selected from the first minute after rTMS. Results showed that there was little difference in power spectra of LFP without PPS between pre-rTMS and post-rTMS signals (Figure 8, red and yellow lines). LFP during PPS showed a decreased power in delta band and increased powers in alpha, beta, and gamma bands (Figure 8, blue lines). The first peak in the PPS power spectrum, which was caused by the fundamental frequency (3–4 Hz) of population spikes during the PPS, and the following peaks caused by the harmonics of the fundamental frequency, formed a signature “sawtooth” pattern of PPS in the power spectrum. These results clearly indicated that the suppression of PPS by rTMS caused the changes of LFP power.

**FIGURE 9:**
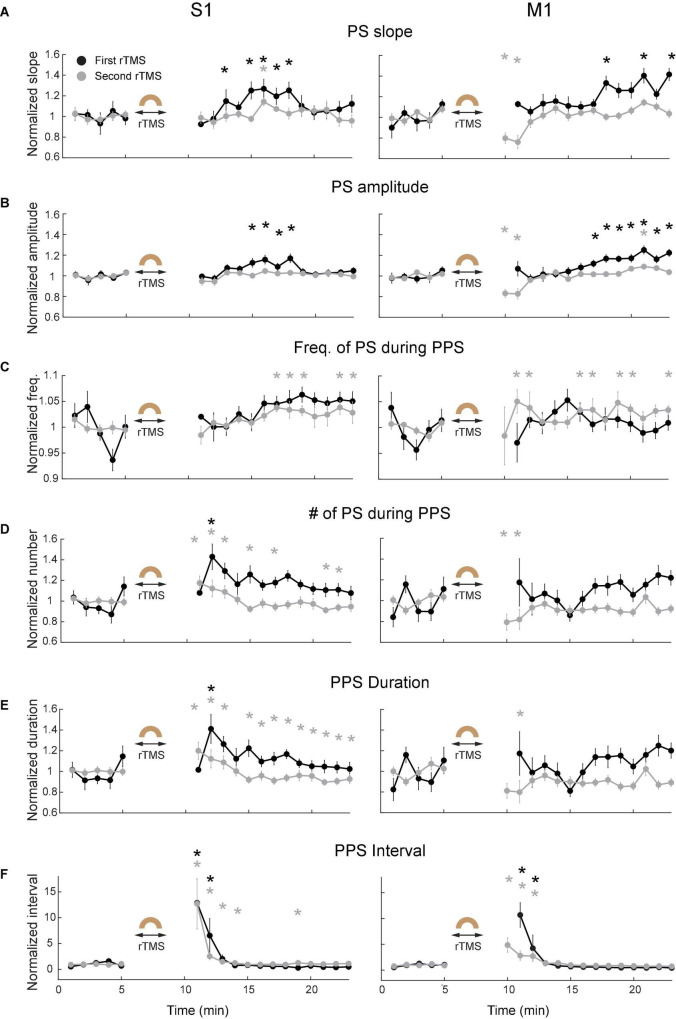
Changes of PPS features after the first (black) and second (gray) courses of rTMS in S1 (left) and M1 (right). **(A)** Slope of population spikes (PS) during PPS. **(B)** Amplitude of PS during PPS. **(C)** Frequency of PS during PPS. **(D)** Number of PS during PPS. **(E)** PPS duration. **(F)** PPS-PPS intervals. All values were normalized and compared with the baseline recordings (*p < 0.05, two-tailed *t*-test). Error bars: SEM.

Since PPS had a non-sinusoidal broadband waveform with canonical oscillation that could not be sufficiently indicated by different frequency bands in its power spectra (Cole and Voytek, 2017), additional waveform-specific metrics such as slope of individual population spike (PS) during PPS, amplitude of PS during PPS, frequency of PS during PPS, number of PS during PPS, PPS duration, and PPS-PPS intervals were used to further quantify PPS features (Figure 9). The slope of PS was measured between the peak of PS and 10 ms before the peak; the frequency of PS in PPS was defined as the mean frequency of PS during a PPS. These metrics were further averaged for every minute in each course of rTMS in both cortices. Results showed that both slope (Figure 9A) and amplitude (Figure 9B) of PS significantly increased several minutes following the recovery of PPS after rTMS in the first course of rTMS (*p* < 0.05). Interestingly, this increase happened ∼5–10 min after rTMS in the S1 but happened ∼10–15 min after rTMS in the M1. Such increase was not observed in the second course of rTMS. This pattern (increase after the first course of rTMS but not the second course of rTMS) was also observed in the frequency bands of LFP (Figure 7). No significant changes were observed in the second course of rTMS (*p* > 0.05) in the S1 except for an increase of PS slope in the eighth minute after stimulation (*p* = 0.0088, *t* = 4.7719). However, the slope and amplitude of PS significantly decreased in the fifth and sixth minutes after the second course of rTMS in the M1 (*p* < 0.05), which might be caused by the gradual recovery of PPS. However, there was no obvious changes during PPS recovery after the first course of rTMS in frequency of PS during PPS (Figure 9C), number of PS during PPS (Figure 9D), PPS duration (Figure 9E), or PPS-PPS interval (Figure 9F), which indicated that the suppressive effects of rTMS on PPS and its recovery were mainly on slope and amplitude of individual PS.

### Repetitive transcranial magnetic stimulation increases spontaneous neuronal spike firing rates

**FIGURE 10:**
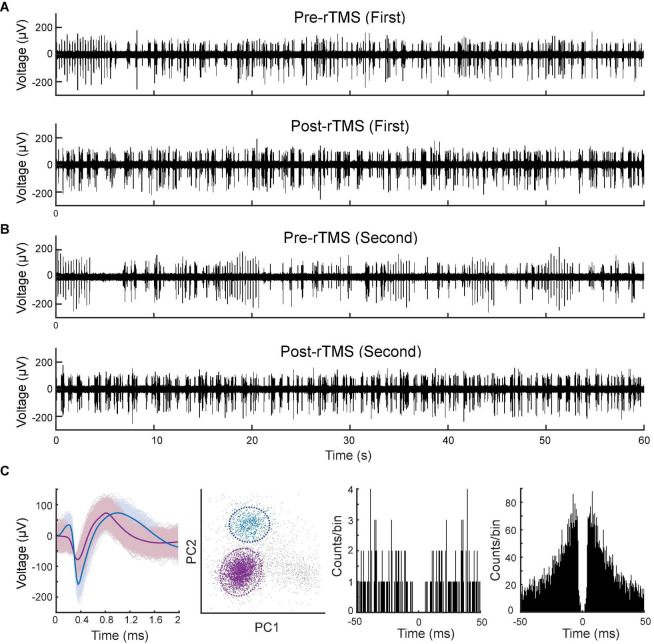
Spontaneous unitary activities recorded pre- and post-rTMS. One-minute of high pass filtered (250 Hz) signals from the M1 before and after the first **(A)** and second **(B)** course of rTMS. **(C)** Waveforms, PCA clusters, and spike autocorrelograms (± 50 ms, bin size: 0.5 ms) of two units sorted from this signal.

**FIGURE 11:**
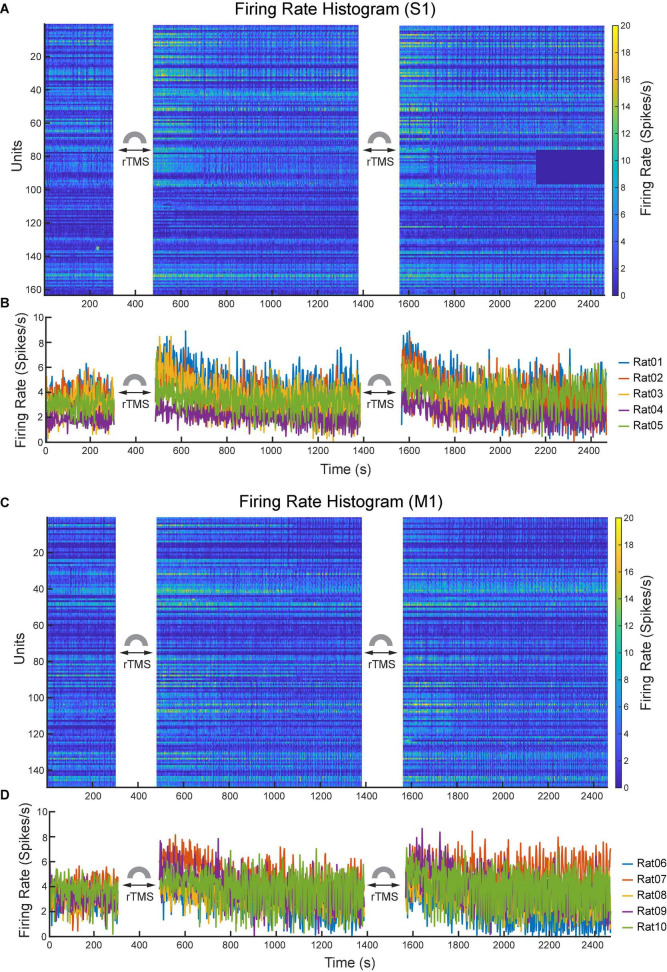
Changes of neuronal spike firing rates (bin size: 2 s) before and after each course of rTMS. **(A)** Firing rate histograms of all S1 neurons (*n* = 163). **(B)** Mean firing rates of S1 neurons from each animal (*n* = 5). **(C)** Firing rate histograms of all M1 neurons (*n* = 149). **(D)** Mean firing rates of M1 neurons from each animal (*n* = 5).

**FIGURE 12:**
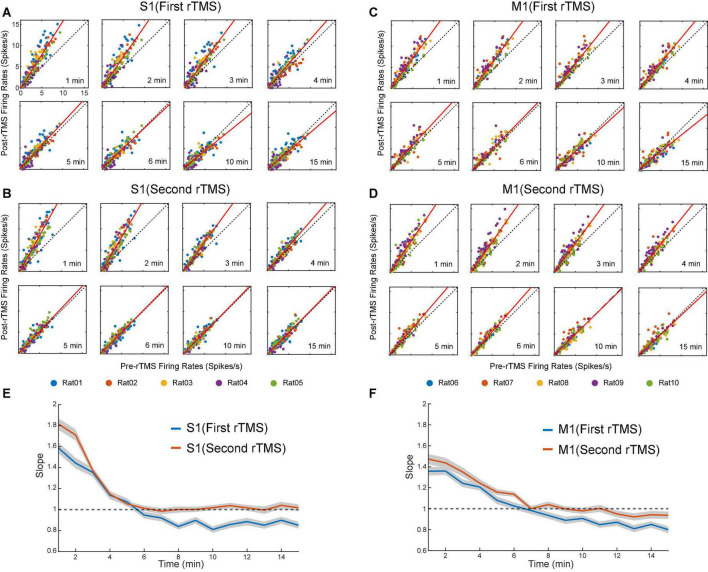
Scatter plots of pre-rTMS vs. post-rTMS firing rates (bin size: 1 min) of neurons at different time points (1, 2, 3, 4, 5, 6, 10, 15 min) after each course of rTMS. Firing rates of S1 neurons are compared before and after the first **(A)** and second **(B)** course of rTMS (*n* = 163). Firing rates of M1 neurons are compared before and after the first **(C)** and second **(D)** course of rTMS (*n* = 149). Linear regression (solid line) is superimposed on the scatter plots. **(E,F)** The slope of the linear fits at each time points after rTMS for both S1 and M1 neurons. Shaded areas: SEM.

Lastly, we compared the single neuron-level activities between pre- and post-rTMS. In total, 163 and 149 single neurons were recorded in the S1 and M1 (*n* = 5), respectively (Table 1). Figure 10 shows a representative example of 1-min-long high pass filtered (250 Hz) unit activities immediately before and after two courses of rTMS (Site63 in Rat06). Both courses of rTMS consistently increased firing rates of neuronal spikes in both S1 and M1 (Figure 11). Different levels of modulation to the single unit activities were shown in each neuron. Linear regressions were performed on mean firing rates of all neurons and compared between pre-rTMS and post-rTMS (Figure 12). Mean firing rates after stimulation were significantly higher than those before stimulation (Figure 12, paired *t*-test, A: *p* < 0.01, B: *p* < 0.01, *n* = 163; C: *p* < 0.01, D: *p* < 0.01, *n* = 149). In both S1 and MI, mean firing rates peaked within 1 or 2 min after rTMS then gradually decayed to a second baseline, which was lower than the first baseline, after the first course of rTMS; while after the second course of rTMS, mean firing rates decayed to the second baseline without further decaying (Figures 12E,F). While both S1 and M1 neurons showed multiplicative increases in mean firing rates in both courses of rTMS, S1 neurons exhibited a more prominent increase in mean firing rates compared to M1 neurons.

**FIGURE 13:**
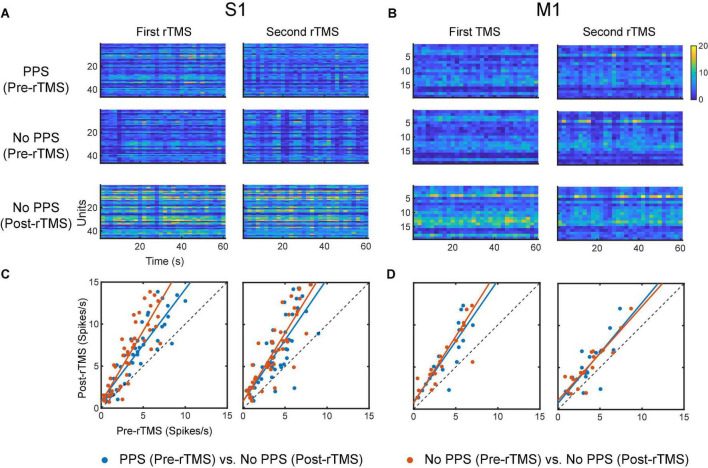
Comparison of neuronal spike firing rates between pre-rTMS signal during PPS, pre-rTMS signal without PPS, and post-rTMS signal without PPS. **(A)** Firing rate histograms of S1 neurons (*n* = 46, bin size = 2 s) during the three types of signals. **(B)** Firing rate histograms of M1 neurons (*n* = 19, bin size = 2 s) during the three types of signals. **(C,D)** Scatter plots of firing rates of neurons. Blue: pre-rTMS during PPS vs. post-rTMS without PPS. Red: pre-rTMS without PPS vs. post-rTMS without PPS.

To evaluate the influence of PPS on the neuronal spike firing rates, firing rates were calculated for pre-rTMS signal during PPS, pre-rTMS signal without PPS, and post-rTMS signal without PPS. In both courses of stimulation, post-rTMS firing rates increased compared to pre-rTMS firing rates despite the occurrence of PPS (Figure 13). Pre-rTMS firing rates during PPS were higher than pre-rTMS firing rates without PPS in S1 neurons but not in M1 neurons.

## Discussion

Most commercially available TMS coils are designed for human subjects and thus have large geometric sizes that cause high-intensity non-focal stimulation in small animals. In addition, those commercially available coils such as classic figure-eight coils cannot be conveniently used in conjunction with the standard MEA due to the limited space between the coil and the brain surface. We have designed, fabricated, and characterized a miniaturized TMS coil for rodent studies. The coil can generate a magnetic field strength (∼400 mT) stronger than those of previously reported TMS coils with similar dimensions (Rodger et al., 2012; Makowiecki et al., 2014; Tang et al., 2016). In both human and animal experiments, motor threshold is determined as the minimum electric field intensity produced by the TMS coil that can result in predefined motor evoked potentials in at least 5 of 10 consecutive trials (Herbsman et al., 2009). The induced electric field generated by our coil was measured to be greater than 3 V/m in a 5-by-3 mm space (at 4 mm) in saline and much lower than the motor threshold of 100 V/m measured in previous rodent TMS studies (Salvador and Miranda, 2009; Boonzaier et al., 2020). However, even at this subthreshold intensity, 10 Hz rTMS, one of the most commonly used TMS protocols, has been shown to increase the amplitude of motor evoked potentials in both humans and rats (Maeda et al., 2000; Tang et al., 2016). To evaluate the reliability and reproducibility of our findings, two courses of subthreshold rTMS at 10 Hz were focally delivered *via* the miniaturized TMS coil to modulate brain activities of the sensorimotor cortex in rats.

The first finding of this study is that ketamine can reliably induce a highly synchronized activities, i.e., PPS, in sensorimotor cortices. To the best of our knowledge, this is the first report of this type of activities in LFP induced by ketamine. The other type of ketamine-induced activity, SWA, is well documented in previous studies (Chauvette et al., 2011; Fiáth et al., 2016; Neske, 2016; Horváth et al., 2021). SWA and PPS shows distinct waveforms and frequency-domain characteristics: SWA is relatively sinusoidal with down-states and up-states. PPS is non-sinusoidal and has highly stereotypical waveforms that consist of multiple population spikes. We found that under deep ketamine anesthesia, SWA, and PPS alternated in a highly regular fashion. This phenomenon presented a novel biomarker of ketamine’s effect on the sensorimotor cortex. Furthermore, due to its high degree of repeatability (observed in all animals), this ketamine-induced PPS can also be used as an animal model for studying cortical oscillation and synchrony in general.

The second finding of this study is that rTMS can effectively and reversibly suppress the ketamine-induced PPS. rTMS changed the power distribution of frequency bands in LFP: it suppressed alpha, beta and gamma bands, while enhanced the delta band, as a direct consequence of its strong suppressive effects on PPS since PPS had high powers in alpha, beta and gamma bands, and lower power in the delta band. After PPS recovery, a “rebound effect” on LFP power was observed in the first rTMS but not in the second rTMS. This “rebound effect” was mainly due changes in slope and amplitude of individual PS during PPS. These changes might be caused by the effect of ketamine alone or the combined effects of rTMS and ketamine. The first possible explanation is that such seeming rebound effect was caused by the gradual increase of ketamine effect alone: ketamine did not reach its full effect before the first rTMS. Therefore, after the suppressive effect of rTMS, ketamine effect kept increasing and eventually reached its steady-state maximum level before the second rTMS. Consequently, such increase happened after the first rTMS but not the second rTMS. If this was what happened, this “rebound effect” was not a real rebound but essentially a summation of rTMS suppression on top of the increase of ketamine effect in the baseline. Previous studies showed that ketamine injection led to dynamic changes in plasma ketamine concentration and brain activities (Sato et al., 2004; Veilleux-Lemieux et al., 2013; Li and Mashour, 2019; Nicol and Morton, 2020). The second possible explanation is that the rebound effect was indeed caused by rTMS; after the short-term suppressive effect of the first rTMS, PPS not only recovered but also rebounded to a higher level of baseline. Such rebound effect should also have been saturated after the first rTMS and thus failed to happen again after the second rTMS. Additional experiments on the pharmacokinetic and pharmacodynamic properties of ketamine and a deeper understanding to the rTMS effect are required to address this question. Nonetheless, the immediate suppressive effect of rTMS to PPS was robust in both courses of rTMS in both cortices.

In addition, we found that SWA and PPS responded very differently to rTMS: rTMS could robustly abolish PPS, whereas rTMS had little effect on low frequency component of SWA. The underlying mechanism of SWA is relatively well understood. It is a synchronized cortical oscillation that involves neurons in multiple brain regions including cortical layers and thalamus (Fiáth et al., 2016; Neske, 2016). Previous studies have suggested that the firing of cortical layer 5 neurons and thalamocortical neurons mainly contributes to the initiation of the up-states; the NMDA receptors may be involved in the persistence of the up-states; the facilitation of inhibitory interneurons, depression of excitatory synapses, or activation of calcium-dependent potassium conductance may result in the termination of the up-states (Neske, 2016). By contrast, PPS induced by high dose of ketamine is a new phenomenon that has never been reported before. Therefore, its mechanisms remain unclear. The distinct effects of rTMS on PPS and SWA suggested that PPS and SWA might be caused by different neuronal and neural network-level mechanisms and these mechanisms could further be differentially modulated by rTMS. The suppression of PPS indicated that rTMS and ketamine had a convergent but opposing effect on synchronized activities in LFP. They may affect the cortical dynamics with overlapping cellular and network-level mechanisms such as the glutamatergic and GABAergic signal transmissions.

Glutamate and GABA are the dominant excitatory and inhibitory neurotransmitters in the central nervous system. Balance between glutamatergic excitation and GABAergic inhibition is crucial for maintaining normal operations of neuronal circuits (Lazarevic et al., 2013; Hampe et al., 2018). It was hypothesized that the administration of ketamine at the anesthetic level might lead to a disruption of GABAergic and glutamatergic systems (Sleigh et al., 2014; Akeju et al., 2016). Blockage of NMDA receptors on interneurons by ketamine reduces GABA signaling and disinhibits pyramidal neurons (Seamans, 2008). Although the mechanism of the ketamine-induced PPS remains unknown, given its similarity in waveforms with interictal spikes, it is likely that the disinhibition of the glutamatergic excitation and the disruption of the GABAergic inhibition contribute to the occurrence of PPS (Avoli et al., 2006; Staley and Dudek, 2006). It must be noted that the effect of ketamine on glutamatergic signaling is dose-dependent. There is an excitatory effect caused by a surge of glutamate following a subanesthetic dose of ketamine, while inhibitory effects occur at an anesthetic dose (Moghaddam et al., 1997; Silberbauer et al., 2020). Interestingly, GABAergic and glutamatergic systems are also potential targets of rTMS. Previous studies have shown that 10 Hz rTMS increases the GABA level in depression patients (Dubin et al., 2016). This increase of GABA may explain the suppression effect of rTMS on the PPS observed in this study. Further experimental and modeling studies are required to fully elucidate the complex interactions of ketamine and rTMS on the cortical oscillations and circuits.

The third finding of this study is that rTMS can significantly increase the firing rates of single neurons in the sensorimotor cortices. The firing rates of neuronal spikes largely increased when there was no PPS, e.g., immediately after rTMS, compared with the pre-rTMS firing rates either during or without PPS. These results suggest that there might be multiple mechanisms contributing to the changes of firing rates caused by rTMS. Indeed, it was also reported that 10 Hz rTMS reduced the GABAergic synaptic strength (Lenz et al., 2016) and increased the glutamatergic synaptic strength (Vlachos et al., 2012; Lenz et al., 2015) in mouse slice cultures. One plausible hypothesis is that the modification of synaptic plasticity caused by rTMS accounts for the increase of spontaneous spiking activities we report in this manuscript. It is worth noting that there is a strong correlation between changes in firing rates and changes in LFP after the first and the second rTMS. It is highly likely that the rebound effects observed in firing rate and LFP power share some common mechanisms, either due to the effect of ketamine along or the combined effects of rTMS and ketamine described above. However, it is unlikely that the changes in firing rate are directly caused by the changes of PPS, since there is no significant difference in firing rates during PPS or periods without PPS (Figure 13). Despite the unknown mechanisms, there seem to be a strong negative correlation between occurrence of PPS, a high-frequency oscillation presumably caused by abnormally synchronized spiking activities, and firing rate of asynchronized spike activities, which presumably underlay normal information processing and brain functions. Our results show that ketamine tends to push cortical circuits to the abnormal synchronized state while rTMS on the other hand is highly effective in restoring cortical circuits to the normal asynchronized state. This opposing effect of ketamine and rTMS on cortical oscillation may have important implications to understanding the underlying mechanisms of ketamine and rTMS.

Neocortical areas utilize similar types of neurons and circuit organizations to achieve different functions (Harris and Shepherd, 2015). S1 is responsible for processing somatic sensations; M1 controls voluntary movements. In this study, rTMS showed similar but variable effects on the S1 and M1: rTMS suppressed PPS for a longer period of time and caused a larger increase of neuronal spike firing rates in the S1 than in the M1. Previous studies have shown that long-term potentiation (LTP) can be more reliably induced by electrical stimulation in the S1 than in the M1 (Castro-Alamancos et al., 1995). Although the rTMS effects showed in this study were much shorter than LTP (minutes vs. hours), it is possible that the two phenomena (stronger responses in the S1 than M1) were caused by similar mechanisms underlying synaptic plasticity.

In this study, the effects of subthreshold rTMS and ketamine on cortical activities were investigated in rats deeply anesthetized with ketamine. The main discovery was that ketamine and rTMS had converging effects on sensorimotor cortical oscillations, and such effects were indicated by the robust induction of PPS by high-dose ketamine and effective abolishment of such PPS by rTMS. It is worth noting that the anesthetic dose of ketamine administered in this study (75 mg/kg) is much higher than the subanesthetic dose used in treating depression and inducing psychedelic effects in humans (0.1–0.75 mg/kg infused intravenously over 40 min) even considering the dose conversion between rat and human (divide by 6.2) (Krystal et al., 1994, 2019; Nair and Jacob, 2016; Andrade, 2017; Ballard and Zarate, 2020). Since the effects of rTMS are inherently state-dependent (Silvanto and Pascual-Leone, 2008), rTMS may produce different after-effects under different doses of ketamine or different anesthesia regimens. Although the discovery of this study could not be directly used to explain the combined therapy of rTMS and subanesthetic ketamine in human studies, it provided a platform for studying the chemical and electromagnetic interactions on cortical circuits at single neuron and neuronal population resolutions. In addition, the alternation of SWA and PPS induced by ketamine observed in this study could be used as a novel biomarker to monitor and characterize the anesthesia state. This phenomenon might provide new insights into the anesthetic action of ketamine on brain activities.

In future studies, we will further investigate the effect of ketamine with different doses and the effect of rTMS with different stimulation parameters in both anesthetized animals and behaving animals performing cognitive tasks. Such studies will deepen our understanding to the underlying mechanism of effects of ketamine and rTMS, as well as their interactions, and may have important implications to the development of a combined chemical and electromagnetic therapeutic strategy for treating neurological and neuropsychiatric disorders.

## Data availability statement

The original contributions presented in the study are included in the article/supplementary material, further inquiries can be directed to the corresponding author/s.

## Ethics statement

The animal study was reviewed and approved by the Institutional Animal Care and Use Committee of the University of Southern California.

## Author contributions

WJ, RI, CL, and DS designed the research. WJ, RI, ZL, and HX performed the research. WJ, RS, ZL, HX, DL, CL, and DS analyzed the data. WJ and DS wrote the first draft. All authors revised and approved the submitted version.

## References

[B1] Akeju O. Song A. H. Hamilos A. E. Pavone K. J. Flores F. J. Brown E. N. (2016). Electroencephalogram signatures of ketamine anesthesia-induced unconsciousness. *Clin. Neurophysiol.* 127 2414–2422. 10.1016/j.clinph.2016.03.005 27178861 PMC4871620

[B2] Andrade C. (2017). Ketamine for depression, 4: In what dose, at what rate, by what route, for how long, and at what frequency? *J. Clin. Psychiatry* 78:10106. 10.4088/JCP.17f11738 28749092

[B3] Avoli M. Biagini G. de Curtis M. (2006). Do interictal spikes sustain seizures and epileptogenesis? *Epilepsy Curr.* 6 203–207. 10.1111/j.1535-7511.2006.00146.x 17260060 PMC1783487

[B4] Ballard E. D. Zarate C. A. (2020). The role of dissociation in ketamine’s antidepressant effects. *Nat. Commun.* 11 1–7. 10.1038/s41467-020-20190-4 33353946 PMC7755908

[B5] Barker A. T. (1991). An introduction to the basic principles of magnetic nerve stimulation. *J. Clin. Neurophysiol.* 8 26–37. 10.1097/00004691-199101000-00005 2019648

[B6] Best S. R. D. Griffin B. (2015). Combination therapy utilizing ketamine and transcranial magnetic stimulation for treatment-resistant depression: A case report. *Int. J. Neurosci.* 125 232–234. 10.3109/00207454.2014.933834 24927244

[B7] Boonzaier J. Petrov P. I. Otte W. M. Smirnov N. Neggers S. F. W. Dijkhuizen R. M. (2020). Design and evaluation of a rodent-specific transcranial magnetic stimulation coil: An in silico and in vivo validation study. *Neuromodulation* 23 324–334. 10.1111/ner.13025 31353780 PMC7216963

[B8] Buzsaki G. Draguhn A. (2004). Neuronal oscillations in cortical networks. *Science* 304 1926–1929. 10.1126/science.1099745 15218136

[B9] Castro-Alamancos M. A. Donoghue J. P. Connors B. W. (1995). Different forms of synaptic plasticity in somatosensory and motor areas of the neocortex. *J. Neurosci.* 15 5324–5333. 10.1523/jneurosci.15-07-05324.1995 7623155 PMC6577900

[B10] Chauvette S. Crochet S. Volgushev M. Timofeev I. (2011). Properties of slow oscillation during slow-wave sleep and anesthesia in cats. *J. Neurosci.* 31 14998–15008. 10.1523/JNEUROSCI.2339-11.2011 22016533 PMC3209581

[B11] Cole S. R. Voytek B. (2017). Brain oscillations and the importance of waveform shape. *Trends Cogn. Sci.* 21 137–149. 10.1016/j.tics.2016.12.008 28063662

[B12] Davila M. C. Hess A. Louviere A. Manzardo A. M. (2021). Ketamine therapy combined with repetitive transcranial magnetic stimulation (rTMS) for major depressive disorder at a suburban tertiary clinic. *Brain Stimul.* 14 1404–1405. 10.1016/j.brs.2021.07.023

[B13] Davis N. J. Tomlinson S. P. Morgan H. M. (2012). The role of beta-frequency neural oscillations in motor control. *J. Neurosci.* 32 403–404. 10.1523/JNEUROSCI.5106-11.2012 22238075 PMC6621073

[B14] Ding L. Shou G. Yuan H. Urbano D. Cha Y.-H. (2014). Lasting modulation effects of rTMS on neural activity and connectivity as revealed by resting-state EEG. *IEEE Trans. Biomed. Eng.* 61 2070–2080. 10.1109/TBME.2014.2313575 24686227 PMC5638649

[B15] Dubin M. J. Mao X. Banerjee S. Goodman Z. Lapidus K. A. B. Kang G. (2016). Elevated prefrontal cortex GABA in patients with major depressive disorder after TMS treatment measured with proton magnetic resonance spectroscopy. *J. Psychiatry Neurosci.* 41 E37–E45. 10.1503/jpn.150223 26900793 PMC4853214

[B16] Engel A. K. Singer W. (2001). Temporal binding and the neural correlates of sensory awareness. *Trends Cogn. Sci.* 5 16–25. 10.1016/S1364-6613(00)01568-0 11164732

[B17] Farmer S. (1998). Rhythmicity, synchronization and binding in human and primate motor systems. *J. Physiol.* 509 3–14. 10.1111/j.1469-7793.1998.003bo.x 9547376 PMC2230956

[B18] Fiáth R. Kerekes B. P. Wittner L. Tóth K. Beregszászi P. Horváth D. (2016). Laminar analysis of the slow wave activity in the somatosensory cortex of anesthetized rats. *Eur. J. Neurosci.* 44 1935–1951. 10.1111/ejn.13274 27177594

[B19] Fries P. Reynolds J. H. Rorie A. E. Desimone R. (2001). Modulation of oscillatory neuronal synchronization by selective visual attention. *Science* 291 1560–1563. 10.1126/science.1055465 11222864

[B20] Hallett M. (2007). Transcranial magnetic stimulation: A primer. *Neuron* 55 187–199. 10.1016/j.neuron.2007.06.026 17640522

[B21] Hampe C. S. Mitoma H. Manto M. (2018). “GABA and glutamate: Their transmitter role in the CNS and pancreatic islets. U: GABA and glutamate-new developments,” in *Neurotransmission research*, ed. Samardzic J. (London: IntechOpen), 65–90. 10.5772/intechopen.70958

[B22] Harris K. D. Shepherd G. M. G. (2015). The neocortical circuit: Themes and variations. *Nat. Neurosci.* 18 170–181. 10.1038/nn.3917 25622573 PMC4889215

[B23] Herbsman T. Forster L. Molnar C. Dougherty R. Christie D. Koola J. (2009). Motor threshold in transcranial magnetic stimulation: The impact of white matter fiber orientation and skull-to-cortex distance. *Hum. Brain Mapp.* 30 2044–2055. 10.1002/hbm.20649 18973261 PMC2893589

[B24] Hong L. E. Summerfelt A. Buchanan R. W. O’donnell P. Thaker G. K. Weiler M. A. (2010). Gamma and delta neural oscillations and association with clinical symptoms under subanesthetic ketamine. *Neuropsychopharmacology* 35 632–640. 10.1038/npp.2009.168 19890262 PMC3055615

[B25] Horváth C. Tóth L. F. Ulbert I. Fiáth R. (2021). Dataset of cortical activity recorded with high spatial resolution from anesthetized rats. *Sci. Data* 8 1–14. 10.1038/s41597-021-00970-3 34267214 PMC8282648

[B26] Jiang W. Isenhart R. Kistler N. Lu Z. Xu H. Lee D. J. (2021). Low intensity repetitive transcranial magnetic stimulation modulates spontaneous spiking activities in rat cortex.in *Proceedings of the 2021 43rd annual international conference of the IEEE engineering in medicine & biology society (EMBC)* (Piscataway, NJ: IEEE), 6318–6321. 10.1109/EMBC46164.2021.9630986 34892558

[B27] Krystal J. H. Abdallah C. G. Sanacora G. Charney D. S. Duman R. S. (2019). Ketamine: A paradigm shift for depression research and treatment. *Neuron* 101 774–778. 10.1016/j.neuron.2019.02.005 30844397 PMC6560624

[B28] Krystal J. H. Karper L. P. Seibyl J. P. Freeman G. K. Delaney R. Bremner J. D. (1994). Subanesthetic effects of the noncompetitive NMDA antagonist, ketamine, in humans: Psychotomimetic, perceptual, cognitive, and neuroendocrine responses. *Arch. Gen. Psychiatry* 51 199–214. 10.1001/archpsyc.1994.03950030035004 8122957

[B29] Lazarevic V. Pothula S. Andres-Alonso M. Fejtova A. (2013). Molecular mechanisms driving homeostatic plasticity of neurotransmitter release. *Front. Cell Neurosci.* 7:244. 10.3389/fncel.2013.00244 24348337 PMC3847662

[B30] Lenz M. Galanis C. Müller-Dahlhaus F. Opitz A. Wierenga C. J. Szabó G. (2016). Repetitive magnetic stimulation induces plasticity of inhibitory synapses. *Nat. Commun.* 7 1–13. 10.1038/ncomms10020 26743822 PMC4729863

[B31] Lenz M. Platschek S. Priesemann V. Becker D. Willems L. M. Ziemann U. (2015). Repetitive magnetic stimulation induces plasticity of excitatory postsynapses on proximal dendrites of cultured mouse CA1 pyramidal neurons. *Brain Struct. Funct.* 220 3323–3337. 10.1007/s00429-014-0859-9 25108309

[B32] Li D. Mashour G. A. (2019). Cortical dynamics during psychedelic and anesthetized states induced by ketamine. *Neuroimage* 196 32–40. 10.1016/j.neuroimage.2019.03.076 30959192 PMC6559852

[B33] Lisman J. (2010). Working memory: The importance of theta and gamma oscillations. *Curr. Biol.* 20 R490–R492. 10.1016/j.cub.2010.04.011 20541499

[B34] Maeda F. Keenan J. P. Tormos J. M. Topka H. Pascual-Leone A. (2000). Interindividual variability of the modulatory effects of repetitive transcranial magnetic stimulation on cortical excitability. *Exp. Brain Res.* 133 425–430. 10.1007/s002210000432 10985677

[B35] Makowiecki K. Harvey A. R. Sherrard R. M. Rodger J. (2014). Low-intensity repetitive transcranial magnetic stimulation improves abnormal visual cortical circuit topography and upregulates BDNF in mice. *J. Neurosci.* 34 10780–10792. 10.1523/jneurosci.0723-14.2014 25100609 PMC4122806

[B36] Mazzeffi M. Johnson K. Paciullo C. (2015). Ketamine in adult cardiac surgery and the cardiac surgery intensive care unit: An evidence-based clinical review. *Ann. Card. Anaesth.* 18 202–209. 10.4103/0971-9784.154478 25849690 PMC4881646

[B37] McCarthy D. A. Chen G. Kaump D. H. Ensor C. (1965). General anesthetic and other pharmacological properties of 2-(o-chlorophenyl)-2-methylamino cyclohexanone HCl (CI-581). *J. New Drugs* 5 21–33. 10.1002/j.1552-4604.1965.tb00219.x 14283065

[B38] Meng Q. Daugherty M. Patel P. Trivedi S. Du X. Hong E. (2018). High-sensitivity and spatial resolution transient magnetic and electric field probes for transcranial magnetic stimulator characterizations. *Instrum. Sci. Technol.* 46 502–518. 10.1080/10739149.2017.1401547

[B39] Meyer R. E. Fish R. E. (2008). “Pharmacology of injectable anesthetics, sedatives, and tranquilizers,” in *Anesthesia and analgesia in laboratory animals American College Of Laboratory Animal Medicine*, 2nd Edn, eds. Fish R. E. Brown M. J. Danneman P. J. Karas A. Z. (San Diego, CA: Academic Press), 27–82. 10.1016/B978-012373898-1.50006-1

[B40] Moghaddam B. Adams B. Verma A. Daly D. (1997). Activation of glutamatergic neurotransmission by ketamine: A novel step in the pathway from NMDA receptor blockade to dopaminergic and cognitive disruptions associated with the prefrontal cortex. *J. Neurosci.* 17 2921–2927. 10.1523/jneurosci.17-08-02921.1997 9092613 PMC6573099

[B41] Mueller J. K. Grigsby E. M. Prevosto V. Petraglia F. W. Rao H. Deng Z.-D. (2014). Simultaneous transcranial magnetic stimulation and single-neuron recording in alert non-human primates. *Nat. Neurosci.* 17 1130–1136. 10.1038/nn.3751 24974797 PMC4115015

[B42] Nair A. B. Jacob S. (2016). A simple practice guide for dose conversion between animals and human. *J. Basic Clin. Pharm.* 7:27. 10.4103/0976-0105.177703 27057123 PMC4804402

[B43] Neske G. T. (2016). The slow oscillation in cortical and thalamic networks: Mechanisms and functions. *Front. Neural Circuits* 9:88. 10.3389/fncir.2015.00088 26834569 PMC4712264

[B44] Nicol A. U. Morton A. J. (2020). Characteristic patterns of EEG oscillations in sheep (*Ovis aries*) induced by ketamine may explain the psychotropic effects seen in humans. *Sci. Rep.* 10:9440. 10.1038/s41598-020-66023-8 32528071 PMC7289807

[B45] Nyhus E. Curran T. (2010). Functional role of gamma and theta oscillations in episodic memory. *Neurosci. Biobehav. Rev.* 34 1023–1035. 10.1016/j.neubiorev.2009.12.014 20060015 PMC2856712

[B46] Paus T. Sipila P. K. Strafella A. P. (2001). Synchronization of neuronal activity in the human primary motor cortex by transcranial magnetic stimulation: An EEG study. *J. Neurophysiol.* 86 1983–1990. 10.1152/jn.2001.86.4.1983 11600655

[B47] Popovych O. V. Tass P. A. (2014). Control of abnormal synchronization in neurological disorders. *Front. Neurol.* 5:268. 10.3389/fneur.2014.00268 25566174 PMC4267271

[B48] Pradhan B. Rossi G. (2020). Combining ketamine, brain stimulation (rTMS) and mindfulness therapy (TIMBER) for opioid addiction. *Cureus* 12:e11798. 10.7759/cureus.11798 33409043 PMC7779150

[B49] Rabiller G. He J.-W. Nishijima Y. Wong A. Liu J. (2015). Perturbation of brain oscillations after ischemic stroke: A potential biomarker for post-stroke function and therapy. *Int. J. Mol. Sci.* 16 25605–25640. 10.3390/ijms161025605 26516838 PMC4632818

[B50] Rodger J. Mo C. Wilks T. Dunlop S. A. Sherrard R. M. (2012). Transcranial pulsed magnetic field stimulation facilitates reorganization of abnormal neural circuits and corrects behavioral deficits without disrupting normal connectivity. *FASEB J.* 26 1593–1606. 10.1096/fj.11-194878 22223750

[B51] Salvador R. Miranda P. C. (2009). “Transcranial magnetic stimulation of small animals: A modeling study of the influence of coil geometry, size and orientation,” in *Proceedings of the 2009 annual international conference of the IEEE engineering in medicine and biology society* (IEEE) (Piscataway, NJ: IEEE), 674–677. 10.1109/IEMBS.2009.5334070 19964482

[B52] Sato Y. Kobayashi E. Hakamata Y. Kobahashi M. Wainai T. Murayama T. (2004). Chronopharmacological studies of ketamine in normal and NMDA E 1 receptor knockout mice †. *Br. J. Anaesth.* 92 859–864. 10.1093/bja/aeh144 15064251

[B53] Seamans J. (2008). Losing inhibition with ketamine. *Nat. Chem. Biol.* 4 91–93. 10.1038/nchembio0208-91 18202677

[B54] Silberbauer L. R. Spurny B. Handschuh P. Klöbl M. Bednarik P. Reiter B. (2020). Effect of ketamine on limbic GABA and glutamate: A human in vivo multivoxel magnetic resonance spectroscopy study. *Front. Psychiatry* 11:549903. 10.3389/fpsyt.2020.549903 33101078 PMC7507577

[B55] Silvanto J. Pascual-Leone A. (2008). State-dependency of transcranial magnetic stimulation. *Brain Topogr.* 21 1–10. 10.1007/s10548-008-0067-0 18791818 PMC3049188

[B56] Sleigh J. Harvey M. Voss L. Denny B. (2014). Ketamine–more mechanisms of action than just NMDA blockade. *Trends Anaesth. Crit. Care* 4 76–81. 10.1016/j.tacc.2014.03.002

[B57] Staley K. J. Dudek F. E. (2006). Interictal spikes and epileptogenesis. *Epilepsy Curr.* 6 199–202. 10.1111/j.1535-7511.2006.00145.x 17260059 PMC1783497

[B58] Steeds H. Carhart-Harris R. L. Stone J. M. (2015). Drug models of schizophrenia. *Ther. Adv. Psychopharmacol.* 5 43–58. 10.1177/2045125314557797 25653831 PMC4315669

[B59] Tang A. D. Lowe A. S. Garrett A. R. Woodward R. Bennett W. Canty A. J. (2016). Construction and evaluation of rodent-specific rTMS coils. *Front. Neural Circuits* 10:47. 10.3389/fncir.2016.00047 27445702 PMC4928644

[B60] Tofts P. S. Branston N. M. (1991). The measurement of electric field, and the influence of surface charge, in magnetic stimulation. *Electroencephalogr. Clin. Neurophysiol.* 81 238–239. 10.1016/0168-5597(91)90077-B 1710973

[B61] Vadivelu N. Schermer E. Kodumudi V. Belani K. Urman R. D. Kaye A. D. (2016). Role of ketamine for analgesia in adults and children. *J. Anaesthesiol. Clin. Pharmacol.* 32 298–306. 10.4103/0970-9185.168149 27625475 PMC5009833

[B62] van Wijk B. C. M. Beek P. J. Daffertshofer A. (2012). Neural synchrony within the motor system: What have we learned so far? *Front. Hum. Neurosci.* 6:252. 10.3389/fnhum.2012.00252 22969718 PMC3432872

[B63] Veilleux-Lemieux D. Castel A. Carrier D. Beaudry F. Vachon P. (2013). Pharmacokinetics of ketamine and xylazine in young and old sprague–dawley rats. *J. Am. Assoc. Lab. Anim. Sci.* 52 567–570. 24041212 PMC3784662

[B64] Vlachos A. Müller-Dahlhaus F. Rosskopp J. Lenz M. Ziemann U. Deller T. (2012). Repetitive magnetic stimulation induces functional and structural plasticity of excitatory postsynapses in mouse organotypic hippocampal slice cultures. *J. Neurosci.* 32 17514–17523. 10.1523/jneurosci.0409-12.2012 23197741 PMC6621866

[B65] Yang J. Liang R. Wang L. Zheng C. Xiao X. Ming D. (2021). Repetitive transcranial magnetic stimulation (rTMS) improves the gait disorders of rats under simulated microgravity conditions associated with the regulation of motor cortex. *Front. Physiol.* 12:587515. 10.3389/fphys.2021.587515 33613305 PMC7890125

[B66] Zmeykina E. Mittner M. Paulus W. Turi Z. (2020). Weak rTMS-induced electric fields produce neural entrainment in humans. *Sci. Rep.* 10:11994. 10.1038/s41598-020-68687-8 32686711 PMC7371859

